# Complying with the framework convention for tobacco control: an application of the Abridged *SimSmoke* model to Israel

**DOI:** 10.1186/s13584-016-0101-8

**Published:** 2016-09-15

**Authors:** David Levy, David B. Abrams, Jeffrey Levy, Laura Rosen

**Affiliations:** 1 Department of Oncology, Lombardi Comprehensive Cancer Center, Georgetown University Medical Center, Washington, DC USA; 2 Department of Health, Behavior and Society, Johns Hopkins Bloomberg School of Public Health, Baltimore, MD USA; 3 Silver Spring, MD USA; 4 Department of Health Promotion, School of Public Health, Sackler Faculty of Medicine, Tel Aviv University, Ramat Aviv, Israel

## Abstract

**Background:**

The World Health Organization Framework Convention for Tobacco Control (FCTC) established the MPOWER policy package to provide practical country-level guidance on implementing effective policies to reduce smoking rates. The *Abridged SimSmoke* tobacco control policy simulation model is applied to Israel to estimate the effects on reducing smoking-attributable mortality resulting from full implementation of MPOWER policies.

**Methods:**

Smoking prevalence from the 2014 Israel National Health Interview Survey 3 and population data from the Israel Central Bureau of Statistics were used to calculate the number of current smokers. The status of current Israeli policy was determined using information from MPOWER 2015 and from local sources. Based on existing knowledge that between 50 % and 65 % of smokers will die prematurely from smoking, the model is used to determine mortality reductions among current smokers from full implementation of MPOWER policies.

**Results:**

We estimate that between 550 and 710 thousand smokers of the current 1.1 million Israeli smokers will prematurely die due to smoking. Within 40 years, complete implementation of MPOWER policies is projected to reduce smoking prevalence among current smokers by 34 % and avert between 188 and 245 thousand deaths among current smokers. Taxes, smoke-free air laws, marketing restrictions and media campaigns each reduce smoking by about 5 % within 5 years. Improved cessation treatment and health warnings each have smaller effects in the next five years, but their effects grow rapidly over time.

**Conclusions:**

Israel *Abridged SimSmoke* shows that complete implementation of the MPOWER strategies has the potential to substantially reduce smoking prevalence, and avert premature deaths due to smoking. Additional benefits are also expected from reduced morbidity, reduced initiation among nonsmokers, and reduction in exposure of nonsmokers to tobacco smoke.

## Background

To reduce non-communicable deaths, the World Health Organization (WHO) set a voluntary target of a 30 % reduction in smoking rates by 2025 as part of its global Non-Communicable Disease agenda [1]. In addition, the WHO launched a policy package that focuses on selected demand side measures for tobacco products, MPOWER, in 2008 [2]. This package recommends the following policies: Monitor tobacco use and prevention policies, Protect people from tobacco smoke, Offer help to quit tobacco use, Warn about the dangers of tobacco, Enforce bans on tobacco advertising, promotion and sponsorship, and Raise taxes on tobacco. The magnitude of the effect of each MPOWER policy on smoking prevalence varies and depends on the policies implemented, how they are implemented, and the country-level policies that were previously in effect [3].

The *Simsmoke Tobacco Control Policy* simulation model has been used to estimate the impact of past policies and potential impact of implementing stronger MPOWER policies on smoking prevalence and smoking-attributable deaths among current smokers within a country or state [4–10]. This model projects the smoking rates over time with the birth and deaths, and the initiation and cessation of smokers. The model predicts well by age and gender for countries that have and have not implemented many strong policies [5–11]. In a previous application [10], we developed a simplified form of *SimSmoke, Abridged SimSmoke,* to evaluate the results of implementing past MPOWER-required policies on country-level reductions in smoking-related deaths. A later paper [12] extended *Abridged SimSmoke* to show the effect of implementing future policies in accordance with MPOWER requirements. The model calculates the impact of the policies on current smokers only, and does not address those who have not yet begun smoking.

The data requirements for *Abridged SimSmoke* are less than for the original *SimSmoke* and parallel the smoking prevalence and policy data collected for the biennial WHO MPOWER/WHO Report on the Global Tobacco Epidemic [1]. Like *SimSmoke*, *Abridged SimSmoke* projects changes in smoking prevalence and smoking-attributable deaths resulting from the implementation of MPOWER policies. We apply that model to Israel.

Israel ratified the FCTC in August 2005 (apps.who.int/fctc/implementation/database/parties/Israel) [13]. Previously, Israel had implemented multiple tobacco policies including taxation (beginning in 1952 and since expanded), smoke-free air policies (beginning in 1982 and since expanded), restrictions on advertising (beginning in 1983 and since expanded), and health warnings (beginning in 1983 and since expanded). Since signing the FCTC, Israel has legislated and implemented stronger smoke-free air laws and began to subsidize cessation treatment through the National Basket of Services. However, despite the passage of the National Plan for Reduction of Smoking and its Harms in 2011, which was based in part on the FCTC and MPOWER, Israel does not yet meet the complete MPOWER requirements for any of the six recommended MPOWER policies [13, 14]. For example, smoke-free regulations are not well enforced [13, 15–20] he planned comprehensive ban on advertising, promotion, and marketing did not pass into legislation, and there is no targeted budget for media campaigns or other tobacco control activities [13].

In this paper, we present the results of the *Abridged SimSmoke* applied to Israel. We estimate the effects of implementing the policies meeting the FCTC-MPOWER requirements on smoking prevalence and smoking-attributable deaths among current smokers in Israel.

## Methods

*Abridged SimSmoke* uses data from a single year to project short-term (5 years) and long-term (40 years) effects on smoking prevalence. It is similar to the original *SimSmoke* in that it relies on population size smoking prevalence and policy modules to make predictions. *Abridged SimSmoke* also uses formulas similar to *SimSmoke* to show a reduction in smoking prevalence in each policy module. Therefore, *Abridged SimSmoke* predicts the effect of individual and combined policies on smoking prevalence and the number of smokers, which is used to project the number of smoking-attributable deaths among smokers alive in 2014.

### Smokers and smoking-attributable deaths

First, the number of smokers, by gender, is obtained by multiplying the respective smoking prevalence and the corresponding population size. Population data for 2014 is from Table 2.5 of the Israel Central Bureau of Statistics. Smoking prevalence (ages 20 and above) is from the most recent nationally representative survey, the 2014 Israel National Interview Health Survey 3, with sample size 2774 [21]. The weighted prevalence rate of smokers was 24.5 % for men and 13.2 % for women.

The number of deaths attributable to smoking is determined using a formula based on the relative risks of smoking. Doll et al [22] concluded that “half of all regular cigarette smokers will eventually be killed by their habit”. Recent studies [23, 24] found that about 65 %, rather than half, of current smoker deaths are attributable to smoking. Upper and lower estimates of deaths expected are calculated by applying the estimates of 50 % and 65 %, respectively. Applying the relevant policy effect sizes, we calculate the expected reduction in smokers and deaths as result of a specific policy or group of policies.

### Policy levels and effect sizes

*Abridged SimSmoke* uses *SimSmoke* policy effect size estimates, which are based on literature reviews, the advice of expert panels, and model validation [4, 5, 10, 25, 26]. For each policy, the effect size, is applied as a percentage reduction in smoking prevalence. An urban adjustor, measured as [1 % employed in agriculture], and the percent not in the labor force, are applied to the work-site effect size, to reflect that smoke-free work-site laws primarily influence the population who work indoors. Israel had 2 % of workers in agriculture [27] and a labor participation rate of 60 % for women and 70 % for men [28].

Based on *SimSmoke*, a long-term multiplier is estimated for each policy as the ratio of the relative change in prevalence (after 40 years) to the relative change in short-term prevalence (after 5 years). This method is applied to the MPOWER policies. These policies are described and their effect sizes listed in Table 1, with upper and lower bound ranges provided in terms of percentage increases and reductions in effect size.

**Table 1:** Policies, specifications and effect sizes used in abridged SimSmoke

Policy	Description	Effect size (% Effect)^a^	Ranges for sensitivity analysis^a^	Long term multi-plier^a^	Urban adjustor^b^
Tax as a percent of retail price of cigarettes	Excise tax, taking into account expanded effect through value added tax	5.9 % (with price elasticity -0.15^c^ and tax as a percent of price increasing from 69 % to 75 %)	(-25 %- + 25 %)	2	no
Smoke-free air laws					
Ban in all indoor workplaces	Ban in all indoor private workplaces	6 %	(-50 %, +50 %)	1.25	yes
Ban in indoor offices only	Ban except ventilated workplaces	4 %	(-50 %, +50 %)	1.25	yes
Ban in health facilities, univ, govt. facilities (2 of 3)	Ban in work areas only	2 %	(-50 %, +50 %)	1.25	yes
Restaurants: Smoke-free in all indoor areas	Ban in restaurants	2 %	(-50 %, +50 %)	1.25	yes
Pubs and bars: smoke-free	Ban in pubs and bars	1 %	(-50 %, +50 %)	1.25	yes
Enforcement	MPOWER: 0-10	25 % of effect depends on % enforcement (of 10)			
Publicity	Based on level of tobacco control funding	25 % of above effect depends on publicity			
Mass Media Campaigns (policies are mutually exclusive)					
Highly publicized campaign	Tobacco control spending ≥ $0.50 USD per capita & media campaign	6.5 % reduction	(-50 %, +50 %)	1.2	no
Moderately publicized campaign	Tobacco control spending ≥ $0.05 and < $0.50 USD per capita	3.5 % reduction	(-50 %, +50 %)	1.2	no
Low publicized campaign	If tobacco control spending < $0.05 USD per capita	1.0 % reduction	(-50 %, +50 %)	1.2	no
Marketing Bans^d^					
Ban on direct and indirect marketing	Ban on all direct and indirect advertising	5 %	(-50 %, +50 %)	1.3	no
Ban on advertising	Ban on all direct advertising	3 %	(-50 %, +50 %)	1.3	no
Partial ban on advertising	Ban on some direct or indirect advertising	1 %	(-50 %, +50 %)	1.3	no
Enforcement	MPOWER: 0 – 1.0	50 % of effect depends on enforcement			
Health Warnings^d^					
Complete	Bold and graphic, covers 50 % of package	2 %	(-50 %, +50 %)	2	no
Strong	Warning 30–50 % of package	1 %	(-50 %, +50 %)	2	no
Weak	Warning <30 % of package	0.50 %	(-50 %, +50 %)	2	no
Cessation Treatment Policies^e^					
Nicotine Replacement Therapy	If sold by pharmacy or general store and if Rx required	Prev. reduced 0.667 % if available at w/out Rx, 0.334 % if Rx	(-75 %, +75 %)	2.5	yes
Bupropion and Varenicline	Sold by pharmacy with prescription	Prev. reduced 0.334 %,	(-75 %, +75 %)	2.5	yes
Provision of treatments	Type facilities: primary care, hospitals, health professionals, community and other	If provided in in most, prevalence reduced 2.25 %, if provided in some, then 1.125 %	(-75 %, +75 %)	2.5	yes
Quit line type	Operating active quit line	Prev. reduced 0.5 %	(-75 %, +75 %)	2.5	yes
Overall effect	With all of the above policies and publicity based on tobacco control funding	Prev. reduced 4.75 %, 25 % of effect depends on publicity	(-75 %, +75 %)	2.5	yes

Notes: *HIC* high-income country, *LIC* low-income country, *MIC* middle-income country, *NA* not applicable, *NRT* nicotine replacement therapy

^a^Short-term effect size is defined as the relative percentage change in smoking prevalence in first five years of policy implementation. The long-term effect is short-term-effect multiplied by the long-term multiplier, adjusted by awareness and urban status adjustors. We also provide ranges for the effect sizes, which are measured as percentage variation in the effect sizes compared to the level in the preceding column

^b^The urban adjustor reduces the effect to reflect the percentage of the rural population not affected by the policies indicated

^c^See Levy et al [32] for a description of the calculations

^d^Categories are mutually exclusive categories

^e^Effects are additive over policies

The effect of implementing stronger policies depends on the initial level of policies. For example, the effect of further marketing restrictions (e.g., in compliance with MPOWER) will be less for a country that already has extensive restrictions than for a country without any marketing restrictions. Data on the level of each policy is from the most recent MPOWER reports [1] and Rosen [13].

Three types of smoke-free air policies (as applied to worksites, restaurants and bars, and other public places) are included in *Abridged SimSmoke*, with the effect of worksite bans further distinguished by their stringency: 1) partial, as designated by a ban in 2 of the 3 following types of facilities: health, university, and government facilities, 2) a ban in indoor offices only, and 3) a ban in all indoor workplaces. The effects are halved in the absence of publicity (based on tobacco control campaign spending as described below) and complete enforcement (an index based on MPOWER reports from 1 to 10, where 10 = complete enforcement). Smoke-free legislation in public places was first introduced in Israel at the national level in 1982 and was expanded over the years to include public transportation, cinemas, theatres, educational institutions, and workplaces, among others; however, the only places to be mandated as 100 % smoke-free were health-care facilities. In 2007, smoke-free legislation (P) shifted from restriction to prevention, with increasing fines and liabilities for owners. In 2012, the ban was extended to youth centers, nursing homes, religious institutions, all government buildings, and some outdoor public areas [13]. Rather than 100 % smoke-free workplaces, smoking is permitted in private offices in non-governmental buildings. In addition, bars, and pubs are permitted to set aside a quarter of their space for smokers as long as it is in a separate room. There are fines for violations on the establishment owners and patrons, but they are inconsistently enforced. When citizen complaints are registered, investigations are undergone but are, likewise, inconsistent in scope and regularity. In Dec. 2015, a new regulation was passed in the Knesset which stipulated that all schools be entirely smoke-free, effective in January 2016. The level of restrictions is set at 50 % for worksites, restaurants and bars and other public places, and compliance is set at level 3 out of 10 in the MPOWER Reports, as it is a well-known problem [19, 20, 29, 30].

MPOWER cessation treatment has three sub-policies: pharmacotherapy (PT) availability, financial coverage of treatments, and quit lines. Quit lines reflect the presence of a national quit line. Israel does not have a national quit line, however, two of its four HMOs have quit lines, and the Israel Cancer Association provides information by telephone. In 2010, smoking cessation technologies were added to the National Basket of Services. Free smoking cessation workshops were made available and Varencline, and Zyban became subsidized, contingent on attendance at the cessation workshops. Nicotine Replacement Therapy, (NRT) which has been available over the counter, was added in 2015 as a second-line medication, in case a smoker was unable to use Varenicline or Zyban. Attempts to add individual and phone counseling have been unsuccessful [13].

Health warnings on packs has four policy levels: none, minimal (<30 % of the principal display area of the pack), moderate (a warning covering at least 30 % of the principal display area of the pack, and meets 1 to 7 of the seven pack warning criteria), and complete (a warning that covers at least 50 % of the principal display pack area and includes all seven pack warning criteria, including graphic warnings, as well as a ban on deceitful terms). According to the 2014 MPOWER Report, Israel has a moderate policy, as the warning covers 30 % of the principal display area of the pack and meets several pack warning criteria. In addition to health warnings, MPOWER includes media campaigns as an education policy. *SimSmoke* rates media campaigns based on the existence of a media campaign and funding levels specified for tobacco control. MPOWER reports do not report expenditures for Israel, but indicate no national campaign. Further, there is currently no dedicated budget for tobacco control [13]. With few staff devoted to tobacco control, media campaigns are categorized as at a low level.

Four levels of marketing restriction policies are designated: none, minimal, moderate, and comprehensive restrictions. They include restrictions on advertising as well as marketing practices, such as branding and sponsorship. For marketing restrictions, no enforcement will reduce the impact of the policy by half. According to the MPOWER Report, Israel has a minimal policy for marketing restrictions, as two out of eight areas are bans on direct advertising and three out of fifteen are bans on indirect advertising, ranked 2 out of 4 (minimal). For direct advertising, Israel has a ban on national TV and radio and fines for violating this ban, but does not have bans on magazines and newspapers, billboards and outdoor advertising, point of sale advertising, and the internet. For bans on indirect advertising, Israel has bans on free distribution in mail or through other means, appearance of tobacco brands in TV and/or film (product placement) and fines for violating these bans, but does not ban promotional discounts, non-tobacco goods or services identified with tobacco brand names, brand names of non-tobacco goods or services used for tobacco products, appearance of tobacco products in TV and/or films, sponsored events, display at point of sale, bans on the tobacco or other industries publicizing their activities, bans on tobacco companies funding or making contributions to smoking prevention media campaigns including those directed at youth, and a requirement to present prescribed anti-tobacco ads before, during, or after the broadcasting or showing of any visual entertainment. According to the MPOWER reports, Israel has advertising restrictions ranked as level 2 out of 4 in the MPOWER Reports. Compliance is at level two of 10.

Cigarette taxation affects cigarette price which, in turn, influences cigarette use. Taxes are specified as a percent of the retail cigarette price. In accordance with MPOWER policies, we consider the effect of increasing excise taxes (including ad valorem taxes or specific (per unit) taxes directly on cigarettes) to 75 % of price. The value added tax (VAT) applies to all goods, not just cigarettes, but amplifies the effect of an excise tax on cigarette price. The change in excise taxes is first translated into the implied percentage change in price. The prevalence elasticity is applied to the percentage change in price to obtain the percent change in prevalence. In Israel, a pack of cigarettes is 30.00 NIS (7.80 USD), of which 17 % is value added (since October 2015) and 69.03 % is excise taxes. There was an increase of over 10 % in cigarette taxes in Israel between 2002 and 2013 [16]. Incorporating the percentage increase in taxes [t/(1 + t)] as amplified through the effect of value added taxes, an increase in taxes from 69 % to 75 % is predicted to increase cigarette prices by 39 %.

The effect of combined policies is calculated with all policies reaching their MPOWER targets. The effects are proportionally reduced for each additional policy. Thereby, relatively conservative assumptions are made about the effects of combined policies (e.g., some overlapping effects), and the overall effect is bounded between zero and one.

However, synergies are built into the model through media campaigns that enhance the effect of smoke-free air laws and cessation treatment policies.

## Results

The results are presented in Table 2. The table first presents the initial levels for smoking prevalence (by gender) and the total number of smokers. Based on the current level of smoking (24.5 % among males and 13.2 % among females), the number of smokers is 1.1 million in 2014. Based on numbers of smokers, the number of smoking-attributable deaths is projected as 550 thousand (350 thousand male and 200 thousand female) as a lower estimate and 710 thousand (455 thousand male and 255 thousand female) as an upper estimate of the smokers alive in 2014.

**Table 2:** Policy effects by MPOWER policy, individual and total: Israel

Current levels	Smoking prevalence		Number of smokers	Projected deaths of smokers (lower)			Projected deaths of smokers (upper)		
	Male	Female		Male	Female	Total	Male	Female	Total
Year 2014	24.5 %	13.2 %	1,093,088	349,468	197,076	546,544	454,308	256,199	710,507
Original policy	Short-term effect size	Long-term effect size	Reduction in number of smokers	Reduction in smoking attributable deaths (lower)			Reduction in smoking attributable deaths (upper)		
				Male	Female	Total	Male	Female	Total
Protect through Smoke-free Air Laws									
Moderate	-4.6 %	-5.8 %	63,403	20,270	11,431	31,702	26,352	14,860	41,212
Offer Cessation Treatments									
Moderate	-2.6 %	-6.5 %	70,918	22,673	12,786	35,459	29,475	16,622	46,097
Mass Media Campaigns									
Low	-5.5 %	-6.6 %	72,144	23,065	13,007	36,072	29,984	16,909	46,893
Warnings on Cigarette Packages									
Moderate	-2.0 %	-4.0 %	43,724	13,979	7,883	21,862	18,172	10,248	28,420
Enforcement of Marketing Restrictions									
Low-moderate	-4.4 %	-5.7 %	62,525	19,990	11,273	31,262	25,986	14,655	40,641
Raise Cigarette Taxes									
Excise Tax 69 %	-5.9 %	-11.7 %	127,969	40,913	23,072	63,984	26,593	14,997	83,180
Combined Policies									
	-22.6 %	-34.3 %	374,408	119,701	67,503	187,204	155,611	87,754	243,365

Notes:

Short-term and long-term effect size are measured in terms of the percentage reduction in smoking prevalence from the initial pre-policy level, i.e., (Post-policy smoking prevalence - Pre-policy smoking prevalence)/Pre-policy smoking prevalence. Lower and upper bounds for the long-term effect size and the reduction in smoking-attributable deaths can be obtained using the ranges for sensitivity analysis from provided in Table 1

Smoking-attributable deaths are based on relative risks from high income nations [22]

The table also shows the effects of each policy individually and in combination. The effects are projected as short and long-term percentage reductions in smoking prevalence and the long-term effects on the number of smokers and smoking-attributable deaths.

- Increasing excise cigarette taxes from its current level of 69 % to 75 % of price will reduce male and female smoking prevalence by 5.9 % within 5 years, increasing to 11.7 % in 40 years, and will avert between 64 and 83 thousand premature deaths.
- Comprehensive smoke-free air laws that made all worksites, restaurants, bars and other public places smoke-free along with stronger enforcement will reduce male and female smoking prevalence by 4.6 % in five years, increasing to 5.8 % in 40 years, and will avert between 32 and 41 thousand premature deaths.
- A well-publicized, comprehensive cessation policy that included a national quitline along with financial full coverage of all treatments will reduce smoking prevalence by 2.6 % in 5 years, increasing 6.5 % in 40 years, and will avert between 35.5 and 46 thousand premature deaths.
- A high level media campaign will reduce smoking prevalence by 5.5 % in 5 years, increasing to 6.6 % in 40 years, and will avert between 36 and 47 thousand premature deaths.
- Pictorial health warnings that cover 50 % of the cigarette package will reduce smoking prevalence by 2 % in 5 years, increasing to 4 % in 40 years, and will prevent between 22 and 28 thousand premature deaths.
- A well-enforced comprehensive ban of direct and indirect advertising (including tobacco products) will reduce smoking prevalence by 4.4 % in 5 years, increasing to 5.7 % in 40 years, and will prevent between 31 and 41 thousand premature deaths.

With the stronger set of policies consistent with FCTC recommendation as specified in MPOWER, the model projects that smoking prevalence can be reduced by 23 % within 5 years increasing to 34 % within 40 years. As a result, between 187 thousand (120 thousand male and 67 thousand female) and 240 thousand (157 thousand male and 88 thousand female) premature deaths of current smokers alive in 2015 are projected to be averted.

## Discussion

Among smokers alive in 2014 in Israel, between 550 and 710 thousand premature deaths are predicted. This demonstrates the urgent need for strong policies to reduce tobacco use. *Abridged Simsmoke* provides estimates of reductions in smoking prevalence, and the resultant number of smoking-attributable deaths which would be avoided, if MPOWER policies were fully implemented [12]. With a complete implementation of policies, Israel is predicted to reach the goal of reducing smoking rates by 22 % in the next 5 years and by 34 % in forty years. As a consequence, between 187 thousand and 243 thousand premature deaths will be averted. The model indicates that increased cigarette taxes, stronger and better enforced smoke-free air laws, and media campaigns and marketing restrictions can play an important role. To a lesser extent, bolder and graphic health warnings and more comprehensive provision of cessation treatments will also contribute to decreasing the burden of tobacco-related mortality.

*Abridged SimSmoke* has been developed based on an extensively validated simulation model and provides support for our estimates. To explicitly consider the predictions of the abridged model, we have compared predictions from *Abridged SimSmoke* for nine countries that have reached MPOWER goals, including Finland and Italy, to results from the complete *SimSmoke* model for or those countries and found that the results predicted by *Abridged SimSmoke* for smoking prevalence and deaths were reasonably close to the reported findings from the complete model [10]. Nevertheless, the findings from *Abridged SimSmoke* should be interpreted in light of the model’s limitations.

*Abridged SimSmoke* does not incorporate future changes in demographics or smoking prevalence that may reflect the effect of previously implemented policies. The abridged model utilizes smoking prevalence data from 2014 as the basis for estimates of the number of smokers and expected deaths due to smoking among current smokers. The model only attempts to incorporate the effects of stronger future policies on those who were smokers in that survey year. It does not incorporate changes in smoking trends, including those that may be influenced by changes in policies implemented prior to that year, or by smoking cessation which may occur spontaneously over time, as indicated by patterns of declining smoking prevalence among older adults [21].

Difference in smoking patterns between the Jewish and Arab population (Age adjusted estimates: Jewish males: 23.1 %, Jewish females: 14.0 %, Arab males: 46.6.%, Arab females: 6.1 %) [21] are not addressed, due to a lack of sufficient information to distinguish differences in relative risks and differences in policy effects among these populations. Further research is warranted.

The model does not incorporate the effects of the unborn, youth, and young adults who will initiate smoking in future years (in the absence of strong policies). Therefore, failure to incorporate cessation of current smokers may be offset by the benefits of newly implemented policies on reducing future initiation. While not considered in our analysis, substantial additional gains can be expected through the effects of policies on the young. In particular, *SimSmoke* attributes more than twice the effect of tax increases on those under the age of 24 as for those ages 25 and above [31, 32]. Further, advertising restrictions are expected to have a 50 % greater effect on those below age 24 [3, 33]. The implementation and enforcement of bans on all smoking in the workplace can also be expected to have an important impact on those newly entering the workforce. These estimates would imply a 25–35 % overall reduction in smoking initiation. We estimate between 550,000 and 710,000 deaths due to smoking. Ginsburg and Geva [34, 35] estimated the number of smoking-attributable deaths at 7,400 per year, implying about 375,000 deaths over 50 years. However, there are important differences between the two methods (e.g., Ginsburg and Geva used deaths by cause vs our use of total mortality, and our upper level estimates are based on higher levels of risks found in the more recent studies [23, 24]). Further study is warranted to examine the composition of deaths by cause and the number of deaths over time by age and gender. In addition, we focused on mortality, but additional costs are associated with morbidity and productivity loss due to premature death. In addition to the 7000 and 7850 smoking-attributable deaths each year, Ginsburg and Geva [34, 35] estimated 319,231 active and 356,601 total smoking attributable hospital days and 1678 million NIS ($482 million) in total health service costs, 0.2 % of GNP. With productivity losses, smoke related costs overall were 3,587 million NIS ($1,030 million), 0.41 % of GNP. We did not include deaths due to second hand-smoke in the model. Globally, secondhand smoke is responsible for 1 % of total mortality and 0.7 % of the worldwide disease burden [36]. We also did not address the economic costs or pain and suffering, caused by tobacco smoke exposure, such as increases in rates of child respiratory disease, low birthweight, sudden infant death syndrome, and pre-term birth [37]. A study of parentally-reported exposure of infants aged 0–2 between 2009–2012 found that 31.5 % (Jewish infants: 24.8 %, Arab infants: 52.0) of infants were at least occasionally exposed to tobacco smoke [21]. A reduction in smoking rates would also decrease exposure of children to smoking.

Another limitation is that the effect of policies is subject to uncertainty. We have provided ranges of the effect sizes in Table 1. In addition, the model only applies to cigarettes and thus does not incorporate other tobacco products such as pipes, cigars, water pipes, and smokeless tobacco. If tax increases and other policies target cigarette smokers exclusively, there may be a substitution toward greater usage of other tobacco products. By directing policies (e.g., media campaigns) at all tobacco products, some of this substitution may be avoided. E-cigarettes potentially provide a lower harm alternative to smoking and therefore may either accelerate or attenuate reductions in net population smoking prevalence depending on how they influence smoking patterns (e.g, initiation and cessation rates) and related policies and practices [38].

*Abridged SimSmoke* has been developed to use data from the biennial WHO MPOWER Reports. The MPOWER policy data are restricted to a specific set of policies and definitions. The model does not consider policies directed at price minimizing behavior [39], enforcement against smuggling [40], product regulation (e.g., nicotine reduction) [41, 42], plain packaging [43, 44], harm reduction policies (i.e., encouraging substitution of less harmful alternatives to cigarettes) [45, 46] and youth access policies [47]. These other policies have also been shown to be effective and are likely to be needed for Israel to approach the endgame for smoking cigarettes.

We did not consider the costs of the interventions relative to their effectiveness. Studies of specific policies have generally found that tobacco control policies are cost effective [48–50]. Taxing policy and health warnings, both of which involve minimal costs, are particularly cost effective. Further study is warranted on the costs of implementation and the cost savings that would specifically be incurred in Israel.

*Abridged SimSmoke* shows that the required MPOWER tobacco control policies will save lives and eventually decrease tobacco use. The model enables the user to consider policies individually and in combination to observe how policies in different combinations lead to differing rates of reductions in smoking prevalence and smoking-attributable deaths. It also translates empirical information into a user-friendly format that can be easily interpreted. This is particularly important given difficulties inherent in tobacco-control policy making, which may include local and global tobacco industry interference [51, 52], lack of public knowledge of the harms of tobacco smoke exposure [12] and underestimation by policy makers regarding public support for smoke-free public places [29, 53].

*Abridged SimSmoke* for Israel shows an urgent need for strengthening comprehensive and proven effective policies and practices to combat and ultimately eliminate the wholly preventable deaths and chronic disease burdens and disabilities caused by tobacco use, primarily driven by the smoking of cigarettes [54, 55].

## Abbreviations

FCTC: Framework convention for tobacco control
MPOWER: WHO package to implement the FCTC
WHO: World Health Organization
