# Erratum to: Complying with the framework convention for tobacco control: an application of the Abridged SimSmoke model to Israel

**DOI:** 10.1186/s13584-016-0121-4

**Published:** 2017-02-08

**Authors:** David T. Levy, David B. Abrams, Jeffrey Levy, Laura Rosen

**Affiliations:** 1 Department of Oncology, Lombardi Comprehensive Cancer Center, Georgetown University Medical Center, Washington, DC USA; 2 Department of Health, Behavior and Society, Johns Hopkins Bloomberg School of Public Health, Baltimore, MD USA; 3 Silver Spring, MD USA; 4 Department of Health Promotion, School of Public Health, Sackler Faculty of Medicine, Tel Aviv University, Ramat Aviv, Israel

## Erratum

There was a minor calculation error in the original version of this paper [1]. The correct numbers are as follows:

Abstract results: The first two sentences should read: “We estimate that between 547 and 711 thousand smokers of the current 1.1 million Israeli smokers will prematurely die due smoking. Within 40 years, complete implementation of MPOWER policies is projected to reduce smoking prevalence among current smokers by 34% and avert between 187 and 243 thousand deaths.”

Results: The 4th sentence in the first paragraph under ‘Results’ on page 5 should read: “Based on numbers of smokers, the number of smoking-attributable deaths is projected as 547 thousand (349.5 thousand male and 197.1 thousand female) as a lower estimate and 711 thousand (454.3 thousand male and 256.2 thousand female) as an upper estimate of the smokers alive in 2014.

Results: On page 7, in the first column the sentence before DISCUSSION should read: “As a result, between 187 thousand (119.7 thousand male and 67.5 thousand female) and 243 thousand (155.6 thousand male and 87.8 thousand female) premature deaths of current smokers alive in 2015 are projected to be averted.

Discussion: The first column, first sentence on page 7 has been changed to: “Among smokers alive in 2014 in Israel, between 547 and 711 thousand premature deaths are predicted.

Discussion: In the first column on page 7, the fourth sentence of the Discussion section has been changed to: “With a complete implementation of policies, Israel is predicted to reach the goal of reducing the goal of smoking rates by 23% in the next 5 years…”

Discussion: On page 7, in the second column: The paper by Ginsberg and Geva is noted three times. Ginsberg has been spelled incorrectly.

Also within the Discussion section, on page 7, second column, middle has been corrected to: “Ginsberg and Geva [34,35]estimated the number of smoking-attributable deaths from active smoking at 7,025 per year, implying about 350,000 deaths over 50 years”

Finally, the following sentence at the end of the second column on page 7 has been amended:

“|In addition to the 7000 and 7850 smoking attributable deaths each year…“ has been replaced with "In addition to the 7,025 smoking-attributable annual deaths due to active smoking and 793 deaths due to passive smoking...."

(Data source: Israel Center for Disease Control) has been added to Reference 21.
